# The Diagnosis of Trichobezoar Based on the Presence of Hypercholesterolemia: A Case Report

**DOI:** 10.7759/cureus.66758

**Published:** 2024-08-13

**Authors:** Toui Tsuchiya, Kohichiroh Nii, Mayumi Yamamoto, Aya Tanaka, Takashi Kusaka

**Affiliations:** 1 Department of Pediatrics, Shodoshima Central Hospital, Kagawa, JPN; 2 Pediatric Surgery, Kagawa University, Kagawa, JPN; 3 Pediatrics, Faculty of Medicine, Kagawa University, Kagawa, JPN

**Keywords:** hypercholesterolemia, pica, trichophagia, trichotillomania, gastric trichobezoar

## Abstract

Trichobezoar refers to the collection of ingested hair accumulating in the gastric mucosa folds, which are found in patients with trichotillomania and trichophagia. We present a case of a nine-year-old girl in Japan who was found to have hypercholesterolemia in the universal lipid screening as a part of the annual health checkups for the prevention of lifestyle-related diseases. Further examination revealed a palpable mass without any digestive symptoms. Imaging studies and the patient's history of pica indicated trichobezoar and upper gastrointestinal endoscopy revealed a large hairball occupying the entire lumen of the stomach. Thereafter, the large trichobezoar was removed by laparotomy and psychological support was provided to her to prevent its recurrence. Hypercholesterolemia improved after the removal of trichobezoar, which suggests an association between a large trichobezoar and cholesterol metabolism. Hypercholesterolemia may serve as a clue to the identification of trichobezoar.

## Introduction

A bezoar is a concrement of indigestible material found in the gastrointestinal tract. Bezoars are classified into four types based on their content: trichobezoar, which is made up of hair; phytobezoar, consisting of undigested fruit and vegetable fibers; lactobezoar, formed from milk; and pharmacobezoar, containing medications [1]. Trichobezoars are predominantly found in young females with psychiatric disorders such as trichotillomania and trichophagia [2]. Therefore, it is important to establish a history of trichotillomania or trichophagia if trichobezoar is suspected. Trichotillomania is characterized by recurrent, compulsive urges to pull out one’s own hair. It most commonly starts in early adolescence. Between 5 and 20% of people with trichotillomania engage in eating the pulled hair, which is called trichophagia.

The repeated swallowing of indigestible hair can lead to its accumulation in the stomach, where it forms a trichobezoar. It is unclear how many patients with trichophagia subsequently develop trichobezoar [3]. When a trichobezoar extends from the gastric body beyond the pylorus into the duodenum, the condition is called Rapunzel syndrome [4]. Trichobezoar is most often detected as Rapunzel syndrome in patients with various digestive symptoms such as vomiting, early satiety, and abdominal pain. We encountered a case where hypercholesterolemia led to the diagnosis of trichobezoar. However, the relationship between trichobezoar and hypercholesterolemia has not been clarified. We report the case and engage in a discussion about this association.

## Case presentation

A nine-year-old girl presented for further examination of her hypercholesterolemia, which had been found during a universal pediatric familial hypercholesterolemia screening in Kagawa, Japan [5]. She did not look pale and had no symptoms of vomiting, abdominal pain, weight loss, or decreased appetite. She had experienced a developmental language disorder at two years of age, but she had not been diagnosed with autism spectrum disorder. At the time of consultation, her mother had a terminal malignancy. Physical examination showed a heart rate of 78 beats/min, a blood pressure of 113/67 mm Hg, and a body temperature of 36.0 ℃. Her height was 125 cm (SD: -1.8), weight was 22 kg (SD: -1.5) and she was 10.0% overweight (normal range: -14% to +19%) with no apparent growth disturbance.

An abdominal examination revealed a palpable, well-defined hard mass (approximately 12 × 9 cm) in the epigastric region. Her mother informed the treating team of a history of trichotillomania, but no patches of hair loss were noted on examination. She also had pica, involving habitual eating of non-nutritional objects such as her nails and chewing gum, this patient's history of pica raised concerns about trichophagia. Blood tests showed no findings suggestive of nutritional disorders such as anemia or hypoalbuminemia, and there were no abnormalities of note except for high total and low-density lipoprotein (LDL) cholesterol levels [221 mg/dL (normal range: 120 - 219 mg/dL; and 150 mg/dL (normal range: 20 - 129 mg/dL], respectively. Thyroid-stimulating hormone and free thyroxin (T4) were normal [1.27 µIU/mL (normal range: 0.5 - 3.5 µIU/mL) and 1.06 ng/dL (normal range: 0.89 -1.53 ng/dL), respectively (Table 1).

**Table 1:** The patient's laboratory data pre- and post-removal of trichobezoar HDL: high-density lipoprotein; LDL: low-density lipoprotein

Laboratory parameter	Patient value pre- and postoperation			Reference range
	Preoperative	At 4 months	At 12 months	
White blood cells (10^3^/µL)	4.9	3.7	3.8	3.8 - 10.1
Red blood cells (10^4^/µL)	491	457	457	407 - 510
Hemoglobin (g/dL)	13.8	13	13.1	11.9 - 14.9
Hematocrit (%)	41.5	39.1	39.3	35.0 - 43.0
Mean corpuscular volume (fl)	84.5	85.6	86	78.0 - 93.0
Platelet (/µL)	35.3	32.1	33	18.0 - 44.0
Total protein (g/dL)	7.3	7.4	6.8	6.2 - 7.9
Albumin (g/dL)	4.5	4.5	4.1	3.6 - 4.7
Aspartate aminotransferase (U/L)	25	22	19	30 - 39
Alanine aminotransferase (U/L)	11	15	9	30 - 39
Lactate dehydrogenase (U/L)	198	177	170	286 - 606
Γ-glutamyl transpeptidase (U/L)	9	12	10	20 - 29
Total cholesterol (mg/dL)	221	204	187	120 - 219
HDL cholesterol (mg/dL)	57	52	53	40 - 99
LDL cholesterol (mg/dL)	150	131	112	20 - 129
Triglycerides (mg/dL)	52	87	63	30 - 139
Urea nitrogen (mg/dL)	10.3	6	7.9	6 - 20
Creatinine (mg/dL)	0.41	0.42	0.44	0.3 - 0.7
Uric acid (mg/dL)	4.1	3.4	3.6	2.1 - 5.9
Blood sugar (mg/dL)	86	97	99	< 100
Hemoglobin A1c(%)	5.3	5.4	5.3	4.8 - 5.5
Thyroid-stimulating hormone (µIU/mL)	1.269	0.81	0.912	0.5 - 3.5
Free triiodothyronine (pg/mL)	3.66	3.18	3.52	1.9 - 4.9
Free thyroxine (ng/dL)	1.06	1.01	0.88	0.89 - 1.53

**Figure 1:**
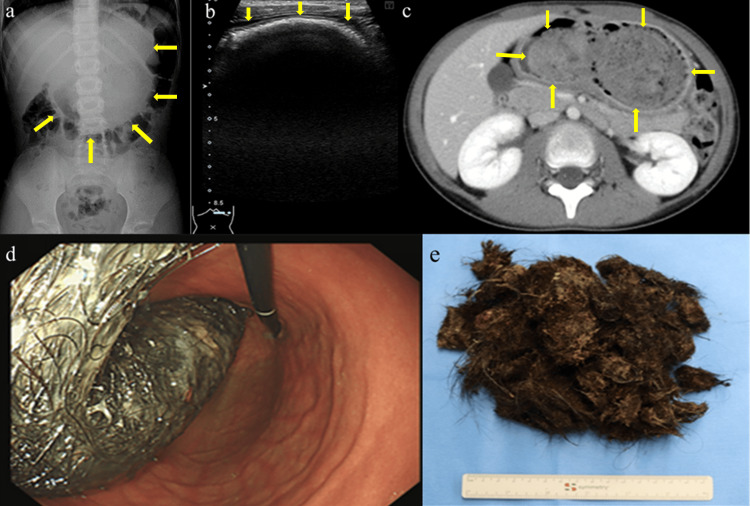
Imaging and macroscopic findings of the trichobezoar (a) An abdominal radiograph shows a mass with a distended stomach and displaced transverse colon. (b) An abdominal ultrasound scan shows an arc-like hyperechoic curvilinear casting with a clear posterior acoustic shadow. (c) Axial contrast-enhanced CT scan showing a well-defined, heterogeneous, and non-enhanced mass. (d) The endoscopic image shows a large, hard, black hairball occupying the entire lumen of the stomach from the fundus to the pylorus. (e) The trichobezoar (201 g) that was removed from the stomach CT: computed tomography

An abdominal radiograph showed the mass in a distended stomach with displacement of the transverse colon (Figure 1a). Abdominal ultrasound showed an arc-like hyperechoic curvilinear casting with a posterior acoustic shadow in the epigastric region (Figure 1b). CT images showed a large, well-defined, heterogeneous, non-enhanced mass in the entire stomach, extending from the gastric fundus to the pyloric canal (Figure 1c). Based on these findings, we suspected that the mass was a trichobezoar. Upper gastrointestinal endoscopy revealed a large, hard, black hairball occupying the entire lumen of the stomach from the fundus to the pylorus (Figure 1d), with no involvement of the duodenum via the pyloric canal, confirming the diagnosis of trichobezoar. The patient was referred to the department of surgery at a tertiary hospital for the removal of the trichobezoar. During laparotomy, a large trichobezoar (201 g) was successfully removed via an anterior gastrotomy incision (Figure 1e).

The postoperative course was uneventful, and the patient was discharged on postoperative day 10 in stable condition. After discharge, the patient has been followed up by the pediatric surgeon and psychiatrist to prevent recurrences of trichotillomania, trichophagia, and trichobezoar. She has not had any additional psychiatric diagnosis. The psychiatrist has conducted counseling sessions along with prescribing Japanese Kampo medicines: yokukansan and kanbakutaisoutou, for irritability.

In addition, we followed up the patient regarding her hypercholesterolemia and found her condition improved. Total and LDL cholesterol decreased to 204 and 131mg/dL four months after surgery. LDL cholesterol improved to 112 mg/dL at the next follow-up one year after the surgery (Table 1).

## Discussion

Based on the upper gastrointestinal endoscopy, it was determined that our patient did not have Rapunzel syndrome. Few cases of trichobezoar detected solely as a palpable abdominal mass, as in this case, have been reported. The differential diagnosis of such masses should include pseudocyst, duplication cyst, and tumor. The unique appearance of trichobezoars in imaging studies distinguishes them from epigastric masses of different etiology [4]. Abdominal ultrasound typically shows a hyperechoic curvilinear casting with an acoustic shadow, and CT scans show a non-enhanced, well-defined, heterogeneous mass with trapped air [6].

Trichobezoar can be suspected based on a history of trichotillomania and trichophagia. In our case, the patient’s pica raised suspicion regarding trichophagia. In some trichobezoar cases, patients habitually ingest inedible material, especially hairlike material such as wool, fibers, or strands, in addition to hair [7]. Trichobezoars can be removed by endoscopy, laparoscopy, or laparotomy. Surgical management is opted based on the patient’s presentation and the size of the trichobezoar. Trichobezoars can remain asymptomatic for many years, but serious complications can result from either this or Rapunzel syndrome, including gastric erosion and ulceration, gastric and small bowel perforation, gastric outlet obstruction, pancreatitis, obstructive jaundice, protein-losing enteropathy, and intussusception, or even death [1]. Early diagnosis and treatment of trichobezoar is therefore important and physicians should be alert for a palpable epigastric mass in patients with histories of trichotillomania and pica.

After the removal of trichobezoar, psychiatric intervention is necessary to prevent the recurrence of trichotillomania and trichophagia [1]. The acts of hair pulling and eating the pulled hair are often accompanied by soothing or pleasurable sensations and the behavior sometimes functions to down-regulate the negative effects. Therefore, trichotillomania often occurs in the context of psychosocial distress and comorbid anxiety, depression, or adjustment problems. The patient’s mother's illness and the patient's trichophagia coincided, and it was inferred that the patient's psychological stress would have contributed to the trichotillomania and the trichophagia, requiring extensive psychological support. No medication with established efficacy and safety is available for trichotillomania. Randomized controlled trials have shown that cognitive behavior therapy is often effective in treating it, with psychiatric consultation and evaluation important for preventing trichobezoar recurrence [7].

Concerning the patient's hypercholesterolemia, which was found at a universal screening test, we observed its improvement after trichobezoar removal. We could not find any data in the literature about the association between trichobezoar and hypercholesterolemia in humans. Interestingly, there is a report on the complication of mild hypercholesterolemia in cats with fabric ingestion [8]. This report suggests that pica and trichobezoar can induce hypercholesterolemia, even though the mechanism behind it is not mentioned. We also presume an association between psychological stress, mild malnutrition with large bezoar, and hypercholesterolemia. It is well known that anorexia nervosa (AN) causes hypercholesterolemia [9].

Several hypotheses have been put forward; hypothyroidism is one of the causes of hypercholesterolemia, which induces a decrease in the fractional clearance of LDL. Ohwada et al. reported an acceleration of cholesterol synthesis in AN with a mild degree of starvation in which the supply of free fatty acid from body fat is not disrupted, even without hypothyroidism [9]. They also concluded that this accelerated cholesterol metabolism is a main pathway for hypercholesterolemia of AN. The coexistence of AN and trichotillomania is very rare; the patient did not have an episode of eating disorder and hypothyroidism either. Although AN was ruled out, mild malnutrition, due to trichobezoar in the stomach, might contribute to hypercholesterolemia.

In the clinical course of our patient, removal of trichobezoar and reduction of cholesterol levels occurred simultaneously; however, no useful information was obtained to suggest a further association. We could not rule out the possibility that the decrease in values occurred due to general guidance related to hypercholesterolemia. The existence of such a confounding factor could not be ruled out. In addition, the interpretation of nutritional disorders had not been adequately evaluated in terms of nutritional indicators including rapid turnover proteins.

## Conclusions

Early diagnosis and treatment of trichobezoar is important to prevent the condition from progressing to Rapunzel syndrome or other serious complications. Physicians should be alert for a palpable epigastric mass in patients with histories of trichotillomania and pica. Hypercholesterolemia may be an additional clue for the detection of trichobezoar.
